# New Insights Into Pathogenesis, Diagnostics, and Therapeutic Options for Canine Angiostrongylosis

**DOI:** 10.1111/vec.70132

**Published:** 2026-09-16

**Authors:** Iris Elgueta, Daniel Lewis, Eric R. Morgan, Jenny Helm

**Affiliations:** ^1^ VetsNow 24/7 Emergency & Specialty Hospital Glasgow UK; ^2^ IVC Evidensia The Chocolate Factory Keynsham UK; ^3^ School of Biological Sciences Queen's University Belfast Belfast UK

**Keywords:** *Angiostrongylus vasorum*, cardiorespiratory, coagulopathy, pathophysiology

## Abstract

**Objective:**

To provide a comprehensive overview of *Angiostrongylus vasorum* infection in dogs, with a particular emphasis on recent developments in the understanding of disease pathophysiology and an update on developments in diagnostic and therapeutic options.

**Etiology:**

*Angiostrongylus vasorum* is a metastrongyloid parasite responsible for varied clinical presentations that primarily include cardiorespiratory, coagulopathic, and neurologic signs. Reports since the turn of the century confirm its continued spread beyond traditional endemic foci, drawing interest in its epidemiology, treatment, and control.

**Diagnosis:**

A definitive diagnosis of angiostrongylosis was historically made with the modified Baermann technique to identify parasite larvae in feces; however, other contemporary diagnostic methods, such as serologic and molecular techniques, are now available to support clinical investigation.

**Therapy:**

Once a diagnosis of angiostrongylosis is made, prompt treatment with anthelmintic drugs (e.g., moxidectin/imidacloprid, milbemycin oxime, fenbendazole) and supportive care should follow, dependent on the patient's clinical signs. This report also provides updates regarding effective prophylactic regimens.

**Prognosis:**

Although angiostrongylosis in dogs can be fatal, and death may be sudden, the prognosis is highly variable and is related to the severity of clinical signs at presentation. Rapid identification and management of the disease with administration of an appropriate treatment can lead to complete clinical resolution.

**Abbreviations**
aPTT: activated partial thromboplastin time
BAL: bronchoalveolar lavage
CT: computed tomography
DIC: disseminated intravascular coagulation
ESPs: excretory/secretory proteins
FFP: fresh frozen plasma
ITS‐2: internal transcribed spacer 2
IPAVAs: intrapulmonary arteriovenous anastomoses
MASP‐1: mannan‐binding lectin serine peptidase 1
MASP‐2: mannan‐binding lectin serine peptidase 2
MRI: magnetic resonance imaging
PCR: polymerase chain reaction
POCUS: point‐of‐care ultrasound
qPCR: quantitative polymerase chain reaction
ROTEM: rotational thromboelastometry
TEG: thromboelastography
tPA: tissue plasminogen activator
TXA: tranexamic acid
vWF: von Willebrand factor

## 1 Introduction

Canine angiostrongylosis, caused by the metastrongyloid nematode *Angiostrongylus vasorum* (Baillet 1866), has gained greater attention recently in small animal practice, likely due to its increasing occurrence around the world and the recognized potential for causing severe disease, leading to fatal outcomes in some cases [1, 2, 3]. *Angiostrongylus vasorum* has an indirect life cycle [4, 5]. Adult parasites reside in the pulmonary arteries and the right side of the heart of their definitive hosts, mainly domestic dogs and foxes. Multiple species of gastropod mollusk (i.e., slugs and snails) act as intermediate hosts [6, 7]. Additionally, frogs and chickens have also been described as paratenic hosts [8, 9].

Dogs become infected after ingesting an intermediate or paratenic host carrying the infective third‐stage larvae (L3). Shedding of L3 by the intermediate host and their survival in the environment have been proven under laboratory conditions, representing a potential additional exposure route; however, the epidemiologic impact and frequency of this occurrence in the field are yet unknown [10, 11]. After ingestion, L3 migrate through the intestinal wall to reach the mesenteric lymph nodes, where they molt into fourth‐stage (L4) and fifth‐stage larvae (L5). L5 subsequently migrate through the liver to the right ventricle and pulmonary vasculature, where they become adult parasites. After mating, ova are released into the pulmonary circulation, where first‐stage larvae (L1) hatch and migrate through the alveolar and bronchial walls. They are then coughed up, swallowed, and excreted in the feces [12, 13]. Importantly, dogs with a subclinical or chronic clinical infection are likely to excrete L1 into the environment for prolonged periods [14, 15, 16].

*Angiostrongylus vasorum* was historically unevenly distributed within Europe, being found in small regions of the United Kingdom, Denmark, Ireland, and France, and was occasionally reported in Spain and Italy, in addition to other long‐standing foci in South America and on the island of Newfoundland off the coast of North America [17]. However, in recent decades, *A. vasorum* has expanded its geographic distribution and has been recognized in countries previously considered free of the parasite [3, 18, 19, 20, 21]. Overall, an increase in the occurrence of *A. vasorum* has been reported, from both endemic and nonendemic regions (Figure 1). The growing concern regarding its geographic range and potential pathogenicity has led to the development of improved diagnostic methods, including new serologic and molecular diagnostic tests [22, 23, 24, 25, 26, 27]. This has enabled confirmation of the expanded distribution of *A. vasorum* in European countries and other parts of the globe via large‐scale seroepidemiologic studies [16, 23, 28, 29, 30, 31, 32]. In the United Kingdom, for example, *A. vasorum* was originally reported in southern regions of England and Wales but has since spread beyond traditional endemic foci [2, 26, 33] and, based on studies in foxes, is now considered endemic across the country [34, 35], with an overall prevalence of up to 3.2% in dogs [32]. Factors hypothesized to be associated with increased distribution and reporting of canine angiostrongylosis include pet travel, climate change, potential of gastropod hosts being moved by “hitchhiking” on horticultural products or other manners, improved diagnostic tests, and awareness and recognition of the disease among practitioners [5, 36]. Foxes, considered the main reservoir of *A. vasorum*, also play an important role in the epidemiology of the disease [34, 35]; in some countries, such as the United States, the parasite is first detected in foxes before cases are seen in dogs [37]. Generally, the parasite has expanded its range into areas predicted to be climatically suitable for its establishment [21], and clinicians should be especially vigilant in these areas even if canine cases are not yet widely recorded.

**FIGURE 1:**
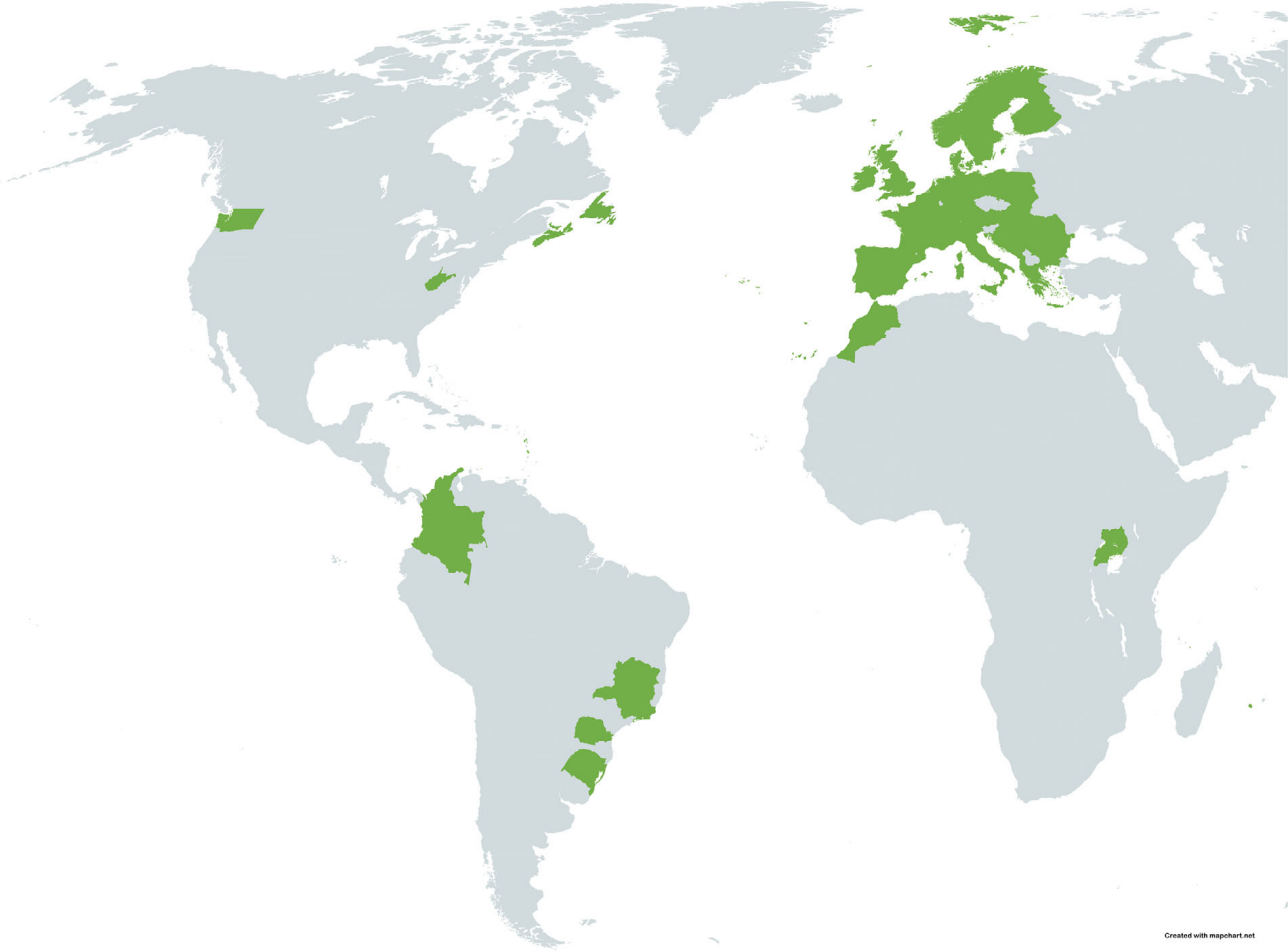
Geographic distribution of reported cases of autochthonous *Angiostrongylus vasorum* in dogs or foxes from 1853 to 2025.

The clinical manifestations and course of disease are highly variable, representing a diagnostic challenge [38] that is exacerbated by unfamiliarity when the parasite spreads to new areas. Respiratory signs are most common, but other manifestations, such as bleeding disorders and, less frequently, neurologic and gastrointestinal signs, with or without respiratory involvement, can occur in infected dogs [4, 15, 39, 40, 41, 42, 43, 44, 45]. Bleeding diathesis is a well‐recognized clinical manifestation observed in some dogs with *A. vasorum* infection [40, 41, 42, 43, 44, 45].

Canine angiostrongylosis has a complex pathogenesis, and many aspects are yet to be elucidated. An understanding of the pathologic mechanisms involved in the development of coagulopathic disorders is currently of great interest. In‐depth evaluations of the coagulation status of dogs infected with *A. vasorum* using viscoelastic methods have been described [42, 43, 44, 45]. Moreover, experimental studies have recently been conducted and have identified alterations of the coagulation cascade and complement system, as well as possible host coagulation interactors released by *A. vasorum* [46, 48].

The aim of this article is to provide a comprehensive overview of clinically relevant aspects of *A. vasorum* infection in dogs, with a particular emphasis on recent developments in the understanding of disease pathophysiology and an update on advancements in diagnostic and therapeutic options.

## 2 Pathophysiology

The pathogenesis of canine angiostrongylosis is complex, and different mechanisms may contribute to the onset of disease. Although dogs infected with *A. vasorum* may present with a wide range of clinical signs, two main clinical syndromes predominate: respiratory disease and bleeding disorders.

### 2.1 Cardiorespiratory Pathogenesis in *A. vasorum* Infection

Respiratory signs are common in canine angiostrongylosis due to the presence of adult parasites and larvae in the cardiorespiratory system [17]. The release of eggs into the pulmonary vasculature and the subsequent migration of L1 through the pulmonary parenchyma induce an intense inflammatory response in the pulmonary arteries and adjacent parenchyma, resulting in verminous pneumonia [4, 17, 49]. Dogs infected with *A. vasorum* typically develop granulomas on the lung surface, arterial wall thickening and thrombosis, pulmonary hemorrhage, and fibrosis of the affected areas [49]. Interestingly, evidence of pneumonia and pulmonary thrombosis has been documented during the prepatent period in experimentally infected dogs [50], highlighting the importance of the development of diagnostic methods to reach an early diagnosis.

Chronically infected dogs with severe lung tissue damage, arterial wall thickening, and thrombus formation are at risk of developing pulmonary hypertension and, infrequently, right‐sided heart failure [40, 51]. Considering the pathogenesis of canine angiostrongylosis, with often severe and extensive pulmonary parenchymal and vascular lesions resulting in increased pulmonary vascular resistance, one would expect pulmonary hypertension to be a common finding. However, pulmonary hypertension has only been reported sporadically and has historically been considered an uncommon complication in dogs naturally infected with *A. vasorum* [51, 52, 53, 54]. Although large studies are lacking, the prevalence of moderate‐to‐severe pulmonary hypertension was 14.6% (*n* = 96) in a referral population of dogs infected with *A. vasorum* [51]. However, not all patients included in the study were evaluated by echocardiography; therefore, cases of mild pulmonary hypertension could have been missed.

When evaluated in an experimental setting, an increased pulmonary arterial pressure was observed over time after inoculation of L3, but only 2 of 6 dogs in this study developed pulmonary hypertension, which was mild with no associated clinical signs [55]. Novo Matos et al. hypothesized the formation of intrapulmonary arteriovenous anastomoses (IPAVAs)—dynamic bypasses created within the pulmonary microcirculation in response to increases in pulmonary vascular pressure, reducing the severity of pulmonary hypertension [53]. These IPAVAs can be found in both physiologic and pathologic conditions and can be detected by saline contrast echocardiography. In the study, IPAVAs were not observed in dogs with severe pulmonary hypertension but were consistently identified in dogs with no or only mild‐to‐moderate pulmonary hypertension, suggesting that the formation of arteriovenous shunts may explain the low prevalence of severe pulmonary hypertension in these dogs [53]. These findings have been documented in a recent case report, supporting the hypothesis that IPAVA recruitment is a protective escape mechanism from pulmonary hypertension, counteracting the increased pulmonary vascular resistance [56]. In the authors’ opinion, however, dogs with severe pulmonary hypertension should have maximal IPAVAs unless there is another mechanism by which they are being closed (e.g., thrombogenesis); therefore, their role in the pathogenesis of angiostrongylosis remains unclear. In contrast to previous findings, a more recent retrospective study found pulmonary hypertension in 58.8% (*n* = 17) of dogs naturally infected with *A. vasorum*, with 35% having moderate‐to‐severe pulmonary hypertension [57]. This study differed from previous studies in that other echocardiographic parameters were evaluated in the absence of tricuspid regurgitation. These findings suggest that pulmonary hypertension may have been underestimated in earlier studies; hence, echocardiographic evaluation should be considered, especially in those cases with marked pulmonary parenchymal changes.

### 2.2 Hemostatic Pathogenesis in *A. vasorum* Infection

Coagulopathic disorders are another well‐recognized pathology associated with *A. vasorum* infection. The mechanisms behind the hemostatic abnormalities induced by *A. vasorum* are incompletely elucidated, and identification of patients at risk of bleeding can be a challenge [44].

Evaluation of coagulation has evolved in the past decades, and new advancements have changed our understanding of the hemostatic system [58, 59, 60]. This led to the development of the cell‐based model of coagulation, characterized by the inclusion of the role of platelets and the cell membrane (Figure 2). Fibrinolysis, the process that regulates the size of the final clot by breaking down fibrin strands, is concurrently stimulated with coagulation when endothelial injury occurs (Figure 2). Fibrinolysis is further enhanced by the generation of thrombin resulting from the activation of the coagulation cascade and is tightly regulated by complex interactions between profibrinolytic and antifibrinolytic processes, preventing excessive thrombosis [61]. Endothelial injury activates the release of tissue plasminogen activator (tPA), which converts plasminogen into plasmin, the major fibrinolytic protease [62]. Plasmin subsequently cleaves fibrin, generating D‐dimers and other fibrin degradation products [63]. Fibrin itself increases the generation of plasmin by binding plasminogen and tPA in the fibrin lysine sites, thus regulating its own destruction. To maintain a balance between clot formation and lysis, downregulation of fibrinolysis is mediated by thrombin‐activatable fibrinolysis inhibitor and serine protease inhibitors, including plasminogen activator inhibitor‐1 and α2‐plasmin inhibitor [62]. Primary hyperfibrinolysis is an inappropriate pathologic response that occurs due to excessive release or activation of tPA or by depletion of plasminogen activator inhibitor‐1, α2‐plasmin inhibitor, and α2‐macroglobulin, leading to activation of fibrinolysis that is systemic rather than localized to the site of the clot [64].

**FIGURE 2:**
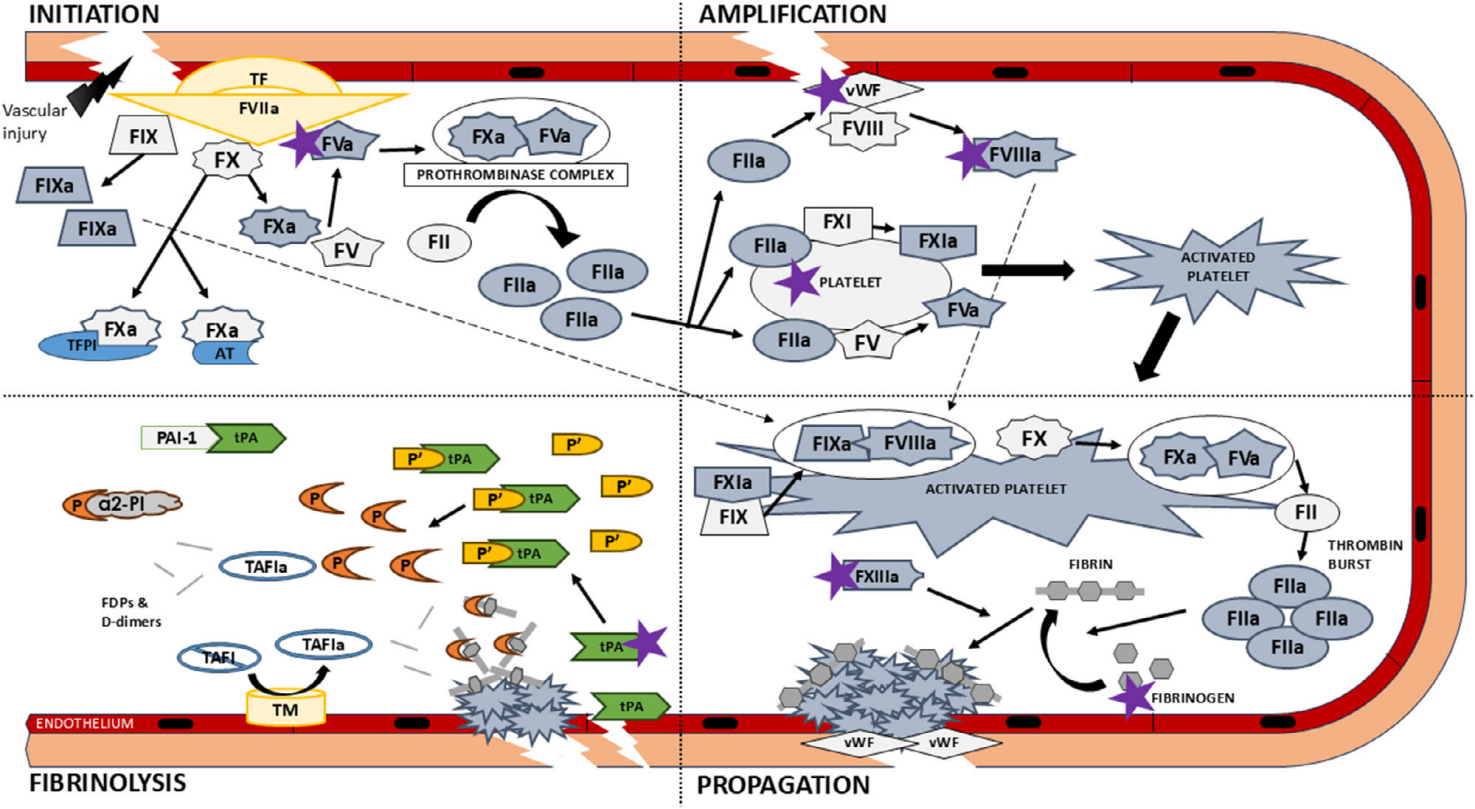
Graphical representation of hemostasis. Hemostasis is initiated after vascular injury, wherein the exposure of TF to circulating blood leads to the rapid binding of FVIIa. In the initiation phase, the resulting TF–FVIIa complex activates small amounts of FIX and FX. FXa is bound to FVa to form the prothrombinase complex, which cleaves FII, generating small amounts of FIIa. During the amplification phase, thrombin induces platelet activation and the activation of FXI, FV, and FVIII after being cleaved from vWF. FXIa further activates FIXa, which then complexes with FVIIIa, constituting the FIXa–FVIIIa complex. This complex enhances FX activation on the surface of the activated platelets during the propagation phase, subsequently binding to FVa and causing the thrombin burst that converts fibrinogen into fibrin and activates FXIIIa, responsible for fibrin crosslinking and stabilization of the fibrin clot. Concurrently, endothelial injury leads to fibrinolysis through the release of tPA, which converts P’ into P. Plasmin degrades fibrin into D‐dimers and other FDPs. Fibrinolysis is tightly regulated by TAFI and serine protease inhibitors, including PAI‐1 and α2‐PI. The purple stars represent some of the possible targets of *Angiostrongylus vasorum*‐induced coagulopathy, discussed in detail in the text. α2‐PI, α2‐plasmin inhibitor; FDP, fibrin degradation product; FII, prothrombin; FIIa, thrombin; FV, factor V; FVa, activated factor V; FVIIa, activated factor VII; FVIII, factor VIII; FIX, factor IX; FIXa, activated factor IX; FIXa–FVIIIa, intrinsic tenase complex; FX, factor X; FXa, activated FX; FXI, factor XI; FXIa, activated factor XI; FXIIIa, activated factor FXIII; P, plasmin; P’, plasminogen; PAI‐1, plasminogen activator inhibitor‐1; TAFI, thrombin‐activatable fibrinolysis inhibitor; TF, tissue factor; tPA, tissue plasminogen activator; vWF, von Willebrand factor.

The hemostatic system can be impaired in different steps of the coagulation process in canine angiostrongylosis, causing abnormalities not only in primary and secondary hemostasis but also alterations in the fibrinolytic system, as documented by more recent studies [44, 45, 65]. Coagulopathies are the second most common presentation of canine angiostrongylosis, with bleeding diathesis being reported in 35%–60% of cases [40, 42, 43, 44, 45]. This prevalence, however, mainly reflects data from referral populations and is likely to represent more severely affected dogs. It is possible that the occurrence of bleeding diathesis is lower in dogs presenting to primary practice [66].

Recent research in canine angiostrongylosis has focused on describing the coagulation status in naturally and experimentally infected dogs and investigating the pathophysiologic mechanisms that may trigger hemostatic dysfunction [42, 43, 44, 45, 46, 47, 48]. Yet the mechanisms behind bleeding disorders induced by *A. vasorum* infection remain under debate. Over the years, multiple hypotheses to explain the pathogenic mechanisms of coagulation disorders in dogs diagnosed with *A. vasorum* have been postulated (Table 1). A low‐grade disseminated intravascular coagulation (DIC) triggered by chronic antigenic stimulation of the hemostatic system has been a popular hypothesis among several studies [13, 43, 67, 68], explained by findings such as variable thrombocytopenia, alterations in secondary hemostasis based on increases in prothrombin time (PT) and activated partial thromboplastin time (aPTT), increases in D‐dimer concentration, and deposits of fibrin in the lungs of dogs infected with *A. vasorum*. However, this theory has been recently questioned and is not supported by some authors [41, 44, 45, 46, 47]. Other possible mechanisms that have been proposed include immune‐mediated thrombocytopenia, factor V and factor VIII deficiencies, acquired deficiencies in von Willebrand factor (vWF), thrombocytopathia, secretion of anticoagulants by the parasite, and activation of the fibrinolytic system [4, 43, 44, 65, 67, 68, 69, 70, 71, 72, 73, 74].

**TABLE 1:** Reported hemostatic abnormalities in dogs infected with *Angiostrongylus vasorum*.

Study	Design	Population (% of clinical bleeding diathesis)	Hemostatic abnormalities
Thomsen et al. [42]	Retrospective cohort study	180 dogs (36.1)	Hypocoagulability (14/55) and hypercoagulability (28/55) on TEG assessment Prolonged PT Moderate thrombocytopenia
Sigrist et al. [45]	Prospective case–control study	18 dogs (39)	Hypocoagulability (9/18), normocoagulability (9/18), and hyperfibrinolysis (8/18) on ROTEM assessment Thrombocytopenia, significantly associated with the presence of bleeding Prolonged PT Increased D‐dimer concentration
Tritten et al. [46]	Experimental study	8 dogs (0)	Lectin pathway downregulation due to decreased MASP‐1 and MASP‐2, and FCN1 ADAMTS13 downregulation, impairing vWF cleavage Coagulation factor XIII‐B depletion Decreased HRG, affecting regulation of both coagulation and fibrinolysis
Gillis‐Germitsch et al. [47]	Experimental study	In vitro	Several ESPs and surface proteins (vWF type D domain protein orthologs, proteases, and protease inhibitors) could interfere with the host coagulation and fibrinolysis
Tayyrov et al. [75]	Experimental study	—	Three unique *A. vasorum* genes encoding folate carrier proteins were identified, with potential involvement in the host coagulation by disrupting host folate–homocysteine metabolism
Sigrist et al. [44]	Retrospective cohort study	21 dogs (57.1)	Hyperfibrinolysis (9/21), often associated with severe hypofibrinogenemia (10/20) Mild‐to‐moderate thrombocytopenia (13/21)
Hausmann et al. [74]	Case report	—	Acquired vWF deficiency and factor VIII deficiency Marked thrombocytopenia
Adamantos et al. [43]	Observational prospective cohort study	30 dogs (60)	Hypocoagulability (22/30) and hypercoagulability (3/30) on TEG assessment Prolonged PT (8/25) and aPTT (12/25) Increased D‐dimer concentration (22/25) Hyperfibrinogenemia (9/18) and hypofibrinogenemia (9/18) vWF antigen: increased (4/25) and decreased (8/25)
Jo'neill et al. [73]	Case report	—	Immune‐mediated thrombocytopenia
Whitley et al. [71]	Case report	—	Acquired vWF deficiency
Chapman et al. [39]	Retrospective case–control study	23 dogs (34.8)	Thrombocytopenia (12/23) Prolonged PT (7/13) and prolonged aPTT (6/13)
Cury et al. [70]	Experimental study	15 dogs	Thrombocytopenia, decreased PT activity and prolongation of PTT, and decreased factor V and VIII activities, suggestive of chronic coagulopathy of consumption
Gould et al. [69]	Case report	—	Immune‐mediated thrombocytopenia
Ramsey et al. [68]	Case report	—	Chronic disseminated intravascular coagulation
Schelling et al. [67]	Experimental study	10 dogs	Thrombocytopenia, prolongation of PT and aPTT, decreased factor V activity, and increased factor VIII activity. These abnormalities were attributed to excessive intravascular coagulation.

Abbreviations: ADAMTS13, a disintegrin and metalloproteinase with thrombospondin type 1 motif 13; aPTT, activated partial thromboplastin time; ESPs, excretory/secretory proteins; FCN1, ficolin‐1; HRG, histidine‐rich glycoprotein; MASP‐1, mannan‐binding lectin serine protease 1; MASP‐2, mannan‐binding lectin serine protease 2; PT, prothrombin time; ROTEM, rotational thromboelastometry; TEG, thromboelastography; vWF, von Willebrand factor.

Recently, the genomic sequence of *A. vasorum* has been studied and compared with other related species, including *Angiostrongylus cantonensis* and *Angiostrongylus costaricensis*, both of which are found to be the most closely related species [75]. Next‐generation sequencing technologies were used to analyze genomic and transcriptomic data from adult female *A. vasorum* nematodes recovered from the lungs of infected foxes. Following phylogenomic analysis, unique *A. vasorum* genes were identified and were considered to be species specific. Of relevance, among the *A. vasorum* exclusive genes, three genes encoding folate carrier proteins were identified [75]. These folate transport proteins are responsible for maintaining a low homocysteine concentration in blood, thus preventing excessive blood clotting. In people, hyperhomocysteinemia due to folate transporter mutations or dysfunction has been shown to lead to formation of fibrin clots abnormally resistant to fibrinolysis, increasing the risk of thrombosis [76]. The identification of these folate carrier genes and their potential involvement in host coagulation by disrupting host folate–homocysteine metabolism is one of the newest insights found in *A. vasorum* infection and provides new targets to explore.

In addition, alterations of the coagulation cascade and complement system have also been described in an experimental study that analyzed the serum proteome in dogs infected with *A. vasorum* [46]. This study identified alterations in several hemostatic proteins, concluding that certain components of the complement system and coagulation pathways appeared to be progressively downregulated over the course of infection. In particular, factor XIII subunit B, mannan‐binding lectin serine peptidase 1 and 2 (MASP‐1 and MASP‐2), a disintegrin and metalloproteinase with thrombospondin type 1 motif 13 (ADAMTS13), and histidine‐rich glycoprotein were found to be affected [46]. MASP‐1 and MASP‐2 are components of the lectin pathway of the complement system, and their activation results in the initiation of the complement cascade. These components are also involved in the cleavage of prothrombin, fibrinogen, factor XIII, and thrombin‐activatable fibrinolysis inhibitor. MASP‐1 and MASP‐2 were significantly decreased in dogs progressing to a chronic phase of the infection (at Day 75 postinoculation), potentially impairing the stabilization of the clot and contributing to the development of bleeding diatheses in these patients [46]. Conversely, ADAMTS13 regulates the interaction between vWF and platelets by cleaving vWF into small inactive fragments, thus limiting platelet activation. A decrease in vWF cleavage due to reduced ADAMTS13 activity during *A. vasorum* infection could, thereby, promote initial platelet plug formation, partially counterbalancing the impaired clot stabilization associated with the downregulation of MASP‐1 and MASP‐2. This group of investigators also characterized the *A. vasorum* excretory/secretory proteins (ESPs) and cuticular surface proteins and evaluated their interactions with the host coagulation process and the immune response [47]. Several hypothetical interactors were released by *A. vasorum*; among these interactors, vWF type D domain protein orthologs, serine proteases, and helminth‐derived protease inhibitors may play a significant role in the pathogenesis of *A. vasorum*–induced coagulopathies [47].

Clinically, a frequently observed finding in dogs with *A. vasorum*–induced coagulopathy is a mild‐to‐moderate thrombocytopenia [4, 39, 42, 43, 44, 45]. Platelet count has been found to be significantly lower in bleeding dogs [42, 45], but this differed from a previous study in which median platelet count was not different between dogs with and without signs of bleeding [44]. Thrombocytopenia appears to be most likely caused by platelet consumption rather than to represent the primary cause of bleeding diathesis, as platelet count rarely decreases below concentrations at which spontaneous bleeding would be expected [42, 44, 45]. Immune‐mediated thrombocytopenia associated with *A. vasorum* infection has been infrequently reported in dogs [69, 73]. To date, platelet dysfunction has not been specifically investigated in canine angiostrongylosis. Nevertheless, rombocytopathia was suspected in dogs showing evidence of thromboelastographic hypocoagulability, with both platelet count and coagulation profile being normal [43]. Although vWF antigen has not been routinely measured in cases of *A. vasorum* infection, in a study by Adamantos et al., a significant difference in this parameter was not identified when comparing bleeding and nonbleeding dogs [43]. Despite this, an acquired von Willebrand syndrome has been suggested in two previous case reports [71, 74].

In an experimental setting, coagulation factor XIII, involved in the crosslinking process to stabilize the fibrin clot, was decreased over the course of infection compared with values before infection [46]. Together with factor V deficiency, also reported in previous studies [67, 68, 70], an increased bleeding tendency may result from reduced clot firmness [46]. Abnormalities in routine coagulation tests (i.e., PT and aPTT) have been reported with variable results in dogs diagnosed with *A. vasorum* infection. While up to 40% of infected dogs had abnormal coagulation profiles in one study, this abnormality was not associated with bleeding diathesis [43]. Therefore, PT and aPTT do not always correlate with the clinical picture, as some dogs with bleeding diathesis may have unremarkable coagulation profiles [4, 39, 43, 45]. The lack of a consistent agreement between PT and aPTT evaluation and clinical signs associated with bleeding diathesis further supports the hypothesis of a multifactorial pathogenesis of bleeding diathesis in canine angiostrongylosis, prompting the need for a better assessment of coagulation in these patients.

Viscoelastic testing, including thromboelastography (TEG) and rotational thromboelastometry (ROTEM), provides a global assessment of the patient's coagulation status, offering a close representation of the in vivo process of clot formation and lysis and detecting both hypercoagulable and hypocoagulable states, as well as hyperfibrinolysis [77, 78]. The study of clot dissolution, however, represents a challenge due to the presence of fibrinolytic inhibitors and activators in whole blood samples [79, 80]. Although viscoelastic methods are considered superior in assessing fibrinolysis compared with the use of D‐dimers [81, 82], these methods may lack sensitivity for the detection of mild‐to‐moderate fibrinolysis [63, 83, 84]. To address these challenges, a modified thromboelastographic assay using recombinant tPA to induce fibrinolysis was developed and standardized in people and has subsequently been used in dogs [85, 86, 87]. The addition of tPA to TEG using whole blood samples has improved the sensitivity for the detection of hyperfibrinolysis; notably, this modified assay was successfully used in a dog infected with *A. vasorum* [63].

TEG and ROTEM have been used in previous studies to describe the coagulation status in dogs naturally infected with *A. vasorum* [42, 43, 45]. Hypocoagulability was identified in 86%–100% of dogs with bleeding diathesis. Of note, up to 25% of dogs with features of hypocoagulability on ROTEM were not exhibiting signs of bleeding [45]. This emphasizes the importance of a thorough assessment of the coagulation status in affected dogs, especially when subtle signs of bleeding could be overlooked during a physical examination. D‐dimer concentrations were frequently increased in dogs infected with *A. vasorum* [42, 43, 45]. These increases result from lysis of crosslinked fibrin, indicating activation of thrombin and plasmin, and are expected to increase with fibrinolysis [82, 88]. Although D‐dimers are a sensitive diagnostic tool for DIC and other thromboembolic disorders, these parameters are poorly specific for these conditions and cannot reliably distinguish them from primary hyperfibrinolysis [89]. A statistical association between D‐dimer concentrations and hypocoagulability was not identified in canine angiostrongylosis in one study [43]. The potential deficiency in factor XIII [46] and possible degradation of fibrinogen and fibrin strands before crosslinking may explain the lack of an association between D‐dimers and hypocoagulability in dogs with *A. vasorum* infection. Hypocoagulability was neither related to antithrombin percentage activity nor fibrinogen concentration, but dogs with evidence of clinical bleeding diathesis had a lower concentration of fibrinogen compared with those without bleeding diathesis [43]. The hypofibrinogenemia identified in these dogs led to the suggestion of primary hyperfibrinolysis as a possible major underlying mechanism of coagulopathy in canine angiostrongylosis [44, 65]. Fibrinogen is a positive acute‐phase protein and is expected to be increased in inflammatory states. However, hyperfibrinolysis, resulting in excessive proteolysis of both fibrin and fibrinogen, may explain the decreased fibrinogen concentration found in *A. vasorum* infection [44]. Severe hypofibrinogenemia associated with hyperfibrinolysis has been previously described in dogs [89, 90]. In a retrospective study of dogs naturally infected with *A. vasorum*, hyperfibrinolysis associated in most cases with hypofibrinogenemia was identified in 67% of dogs with clinical signs of bleeding [44]. A subsequent prospective study reported hyperfibrinolysis in 56% of dogs with clinical bleeding diathesis secondary to *A. vasorum* infection [45]. A larger retrospective study documented the presence of hyperfibrinolysis based on TEG in 13 of 33 dogs (39%) with bleeding diathesis, contrasting with 3 of 22 dogs (14%) without signs of bleeding [42]. The tPA‐modified TEG was not used in this study, which may have reduced the ability to detect hyperfibrinolysis.

Overactivation of the fibrinolytic system in canine angiostrongylosis is thought to be multifactorial. Hyperfibrinolysis may be induced by the pathogen itself after endothelial trauma, leading to mechanical or chemical injury of the endothelial cells within the pulmonary arteries and stimulating the release of tPA and subsequent generation of plasmin [44]. Additionally, *A. vasorum* may similarly interfere with the host fibrinolytic system as reported in dogs infected with *Dirofilaria immitis*, in which excretory/secretory antigens from adult *D. immitis* worms have been shown to activate fibrinolysis by enhancing tPA expression and binding to plasminogen [91, 92]. Evaluation of the effect of *A. vasorum* ESPs on dog coagulation and fibrinolysis by ROTEM was carried out in vitro on canine endothelial cells [47]. This study revealed that the interaction of ESPs with host vascular hemostasis was minimal and that the onset of fibrinolysis was not influenced by ESPs, and the authors concluded that *A. vasorum* ESPs and surface proteins are not solely responsible for bleeding in dogs, at least in the acute phase of the infection [47].

It is also possible that prolonged exposure to *A. vasorum*, as it occurs in naturally infected dogs, could lead to the release of procoagulant components or profibrinolytic factors capable of modulating the host vascular hemostasis [47]. A more recent study using N‐terminomics profiling to identify host proteins targeted by *A. vasorum* ESPs proteases showed that eight dog proteins were significantly cleaved upon exposure to the parasite's ESPs, including coagulation factors (II, V, and IX) and complement proteins (C3, C4‐A, and C5) [48]. This finding may suggest a direct or indirect interaction of *A. vasorum* ESPs with host coagulation and complement cascades.

The use of viscoelastic testing has allowed the detection of hypercoagulability in these cases. Although not frequently documented, thromboelastographic hypercoagulability was recently identified in 50.9% of dogs diagnosed with *A. vasorum* [42]. Of the hypercoagulable dogs, 42.9% were also exhibiting signs of bleeding. This finding is relevant, especially regarding the therapeutic management of these patients. Large prospective studies using viscoelastic methods are still needed to estimate the incidence of hypo‐ and hypercoagulability, as well as hyperfibrinolysis, in dogs infected with *A. vasorum*, with and without clinical bleeding diathesis.

## 3 Clinical Presentation

Dogs infected with *A. vasorum* can present with an array of clinical signs. The clinical course of disease is highly variable, ranging from subclinical cases or chronic presentations to acute life‐threatening manifestations with often fatal outcomes [1, 2, 3]. Canine angiostrongylosis has been reported in dogs of all ages; however, younger dogs, especially under 1 year of age, seem more likely to become infected [4, 39, 41, 42]. The reasons for this could be because younger dogs have higher scavenging behavior compared with older dogs [5] or due to their higher propensity to develop severe signs as a result of immune incompetence [39, 93]. Besides an individual's immunologic state, the variability in the severity of the clinical signs may also reflect worm burden, as documented in experimental studies [94]. Some studies have documented an overrepresentation of the disease in certain breeds, including the Cavalier King Charles Spaniel and Staffordshire Bull Terrier [4, 39, 43]; however, a breed predisposition has not been further established.

Due to the primary localization of the parasite, the classic clinical signs of canine angiostrongylosis are cardiorespiratory in nature, predominantly presenting with cough and dyspnea secondary to verminous pneumonia. Respiratory signs have been reported in 52%–65% of dogs infected with *A. vasorum* [38, 39, 40]. In more chronic cases, severe pulmonary hypertension and congestive right‐sided heart failure can occur, manifesting as weakness, exercise intolerance, syncopal episodes, or ascites, in some cases leading to a fatal outcome [52, 53, 54]. Of note, although respiratory signs are common, almost half of the infected dogs in one study did not present with respiratory signs, and over one‐third of cases were asymptomatic [38], raising the concern that some of these cases may be overlooked by practitioners less familiar with the disease.

Bleeding disorders represent the second most common reported manifestation of *A. vasorum* infection. When comparing studies, there is wide variability in the percentage of dogs displaying bleeding diathesis, ranging from 5.6% to 71% [38, 40]. The higher proportion may be biased by the type of population included in the study [40] and likely reflects an increased likelihood of referral in those patients presenting with bleeding diathesis, whereas the lower proportion of reported cases could be due to unrecognized signs of minor bleeding [38]. Overall, the most frequent prevalence of coagulopathies associated with *A. vasorum* infection reported in the literature has been between 35% and 60% [39, 42, 43, 44, 45]. Coagulopathies may result in a variety of manifestations, including petechiae, ecchymoses, hematomas, scleral hemorrhage, epistaxis, hemoptysis, postsurgical bleeding, gastrointestinal bleeding, and hematuria [5, 39, 40, 41, 95, 96]. Hemoabdomen and hemothorax have been reported in previous cases [72, 97]. One study evaluating dogs with unexplained bleeding, eventually diagnosed with *A. vasorum* infection, found that half of the cases did not show signs of respiratory disease, nor did they have thoracic radiographs suggestive of angiostrongylosis [41]. This has been a clinical feature in other studies as well; therefore, the absence of respiratory signs in affected dogs does not exclude *A. vasorum* infection and emphasizes the importance of this disease being included in the differential diagnosis of any dog presenting with unexplained bleeding tendencies [38, 39, 40, 41, 42].

Intracranial and intraspinal hemorrhage has also been described in canine angiostrongylosis [98, 99, 100, 101, 102]. One large retrospective study found that >10% of dogs presented with neurologic signs associated with bleeding diathesis in the CNS [42]. A few case reports have described an ocular manifestation of the disease that presents with scleral, subconjunctival, iridal, and retinal hemorrhage, hyphema, or uveitis [26, 38, 103]. Sporadically, aberrant larval migration to multiple organs and the presence of adult worms in atypical locations have also been reported [103, 104, 105, 106]. In addition, pneumothorax has been occasionally associated with *A. vasorum* infection [107, 108].

Mild and nonspecific clinical signs like lethargy, anorexia, weight loss, or gastrointestinal signs, with or without concurrent respiratory signs, may also be seen in infected dogs [38, 39]. While this presentation is less commonly reported, such milder presentations may be more common in primary practice. Almost 39% of dogs presenting for a routine check‐up in which *A. vasorum* L1 was identified in feces using the Baermann technique had nonspecific clinical signs [38]. Of those dogs, only half had concurrent cardiorespiratory signs.

Physical examination findings vary depending on the clinical presentation. Although thoracic auscultation can be unremarkable, more severely affected dogs may present with tachypnea and have abnormally increased lung sounds or crackles on auscultation [4, 39, 49]. A heart murmur may be identified in some cases [51, 52]. A thorough examination of the skin and mucous membranes should be performed, specifically looking for evidence of hemorrhage. Occasionally, infected dogs may present with pyrexia [39], but this can be intermittent during the course of infection [49].

Hematologic and biochemical changes can be mild to moderate, but no pathognomonic signs are specific for *A. vasorum* infection. Eosinophilia was reported in 39% of infected dogs presented to a referral institution [39], but this was in contrast with other reports in which eosinophilia was identified less frequently [40, 66]. Neutrophilia, anemia, and thrombocytopenia are also commonly reported hematologic abnormalities [39, 66]. Biochemical changes tend to be mild, with hyperglobulinemia being one of the most consistent findings [66]. Increased alkaline phosphatase, alanine aminotransferase, and creatine kinase activities, hyperbilirubinemia, and hypoalbuminemia have also been documented [39, 66]. Hypercalcemia thought to be secondary to granuloma formation has been sporadically described in canine angiostrongylosis [109]. Measurement of ionized calcium in dogs infected with *A. vasorum* is indicated, especially if clinical signs consistent with hypercalcemia, such as polyuria and polydipsia, are present. Another clinicopathologic abnormality observed rarely in canine angiostrongylosis is a low serum fructosamine concentration [110].

## 4 Diagnosis

The highly variable clinical presentation of canine angiostrongylosis may make it challenging to consider as a differential diagnosis. A definitive diagnosis is made upon confirmation of the presence of the parasite or an immunologic response to it. The disease's continued geographic expansion may lead practitioners to overlook the clinical signs suggestive of angiostrongylosis and to treat patients for other similarly presenting conditions [41, 111], resulting in delays in diagnosis and institution of appropriate therapy. Development of severe pathologic pulmonary changes has been reported even in the absence of clinical signs [49, 50]; therefore, an early diagnosis is essential. To that end, research has been focused on developing molecular and serologic assays to improve the speed and accuracy of diagnosis [22, 25, 26, 27, 112, 113].

### 4.1 Anamnesis

Despite recent expansion in range, the distribution of *A. vasorum* remains rather focal, and being in an endemic area or one at risk of parasite colonization, or having travelled to one, is an important factor in differential diagnosis. Other risk factors, especially young age, are also relevant [15]. Effective measures to prevent infection during the prepatent period, such as prophylactic anthelmintic drugs against *A. vasorum*, can reduce parasite establishment. Licensed preventive regimens are currently administered monthly [114, 115, 116, 117, 118], and although less frequent worming treatment or monthly treatment with unlicensed anthelmintics probably reduces the risk of infection, it does not eliminate the possibility [15]. The absence of an effective chemoprophylaxis regimen in dogs with other risk factors should therefore increase the suspicion of angiostrongylosis.

### 4.2 Identification of First‐Stage (L1) Larvae

Traditionally, canine angiostrongylosis has been diagnosed based on detection of L1 recovered from feces using the modified Baermann technique [4, 39]. In this test, feces are submerged in water, and L1 actively migrate out of the feces and across a mesh, sinking and subsequently being recovered onto a microscope slide [17]. One advantage of this test is that it allows morphologic differentiation from other lung worm larvae, such as *Crenosoma vulpis* and *Filaroides* spp [119]. Skilled personnel are required to correctly identify the morphologic features of *A. vasorum* larvae, although a presumptive diagnosis by informed nonspecialists is reasonable based on the recovery of multiple vigorously moving nematode larvae with a sinusoidal tail projection and characteristic dorsal spine. The modified Baermann technique has been one of the most common methods used to diagnose canine angiostrongylosis, but its sensitivity is limited, especially when performed on a single fecal sample. Moreover, this test will fail to detect L1 during the prepatent period of *A. vasorum* infection in patients that are already clinically affected [49]. Generally, the prepatent period varies between 33 and 76 days, as determined by experimental studies [12, 14, 49, 120]. False‐negative results may also occur due to the intermittent shedding of L1 and the variable parasite numbers in feces during the infection [14]. Analysis of fecal samples collected over 3 consecutive days has been recommended to increase the diagnostic sensitivity; however, a recent study showed that larval migration is considerably decreased over the course of 3 days of sample storage. Hence, the authors questioned the value of this approach and instead proposed collecting several fecal samples for 1 day [121]. The cumbersome nature of the test and the length of time to obtain results if sent to an external laboratory can make this test impractical in some clinical settings. In contrast, a direct fecal smear can be useful to support a rapid diagnosis of angiostrongylosis, with a reported sensitivity of up to 61% when compared with the Baermann technique [122]. Although fecal smears are quick and inexpensive as a bedside test, they can have a suboptimal sensitivity, especially when the test is performed by inexperienced staff. Fecal flotation methods used for the detection of parasite ova, oocysts, and larvae from fecal samples are also likely to have low sensitivity, although more recent methods such as FLOTAC were found to be more sensitive than the Baermann technique in detecting L1 after prolonged sample storage [123]. Ultimately, a negative fecal sample by any of these methods does not exclude the disease, and diagnostic techniques more sensitive than fecal examinations should be performed if the clinical suspicion of angiostrongylosis is high [113, 124, 125].

Tracheal wash or bronchoalveolar lavage (BAL) fluid analysis is an alternative method to identify L1 [39, 120, 124]. BAL is an invasive technique, however, and should be weighed against the risks, especially in patients with compromised respiratory function. Generally, though, it is considered a safe procedure with a low incidence of postprocedural complications [126]. It also has the advantage of allowing cytologic and microbiologic evaluation of the airway, providing additional diagnostic information relevant to the therapeutic management of the patient. In dogs experimentally infected with *A. vasorum*, L1 were detected on cytologic evaluation of BAL fluid before fecal larval shedding, suggesting that BAL fluid analysis may allow an earlier diagnosis [120]. However, this has not been a consistent finding in naturally infected dogs [39]. A relatively increased number of neutrophils and eosinophils in BAL fluid has been documented in infected dogs [120], but the predominant finding on cytologic examination is a neutrophilic inflammation with infrequent bacterial involvement, with a mixed neutrophilic and eosinophilic inflammation being less common [39, 124]. Other diagnostic methods for the identification of L1 have been sporadically described, including the detection of L1 in fine‐needle aspirates of a consolidated lung lobe [127], in urinary sediment through microscopic examination [4], and in pleural effusion [72]. Dissemination of larvae across multiple tissues has been described and associated with pathology [1, 100] and could presumably be identified through histopathologic biopsy or postmortem material.

### 4.3 Serologic and Molecular Methods

Because of the limitations of the fecal techniques in detecting dogs with *A. vasorum* infection, blood tests and isolation of parasite DNA from different substrates have been developed to overcome these problems. The ease of obtaining an adequate sample and the usefulness for both large‐scale epidemiologic studies and individual diagnosis of angiostrongylosis make blood‐based tests attractive.

Initially, investigations were carried out to identify stage‐specific antigens recognized by antibodies [112, 128]. This allowed specific antibodies and circulating *A. vasorum* antigens to be detected using ELISA, tests that have been successfully validated for the diagnosis of canine angiostrongylosis [23, 25]. A sandwich ELISA was first developed using polyclonal antibodies for the detection of circulating *A. vasorum* antigens [113]. This sandwich ELISA, tested in sera of dogs naturally infected with *A. vasorum*, showed promising results with a reported sensitivity of 92% and a specificity of 100%, although low parasite burdens were suspected to result in false‐negative results. No cross‐reactivity against several helminths, including *C. vulpis*, was found; however, cross‐reaction against *D. immitis* was not evaluated, which could have led to a lower specificity. This was clinically relevant because *A. vasorum* and *D. immitis* can be found in similar geographic locations and may share common epitopes, potentially inducing cross‐reactions [22, 129].

Interestingly, the Baermann technique failed to detect 23 of the 47 (49%) infected dogs in this study, whereas the sandwich ELISA identified 45 of the 47 infected dogs, assuming that the positive results truly detected dogs with *A. vasorum* infection [113]. Subsequently, a new ELISA was described using both species‐specific monoclonal antibodies, selected to exclude cross‐reactions with *D. immitis*, and polyclonal antibodies directed against *A. vasorum* adult excretory/secretory antigen [22]. The sensitivity and specificity of this ELISA test were 95.7% and 94%, respectively. Antibody‐based ELISAs, along with detection of the parasite DNA in blood by means of molecular methods, represent the quickest diagnostic tools in detecting infection even before patency [25, 130]. It is worth noting, however, that the presence of antibodies in dogs previously infected could lead to false‐positive results as these tests may remain positive for several weeks after treatment [130]; therefore, antibody‐based assays should not be the only diagnostic tool used in the clinical setting, especially in endemic areas where the exposure to *A. vasorum* could be high.

These developments allowed for the design of a lateral flow immunochromatography test^1^, which detects circulating *A. vasorum* antigen and, relative to the Baermann technique, provides an alternative and rapid identification of infected dogs for in‐clinic diagnosis [24] with moderate sensitivity (85%–97.1%) and high specificity (98.9%–100%) [24, 130, 131]. This commercially available test can detect circulating antigens as early as 9 weeks following experimental inoculation of L3 in dogs and can be used for monitoring purposes after appropriate anthelmintic therapy [24, 111]. Nonetheless, this method will not detect infection during the prepatent period and, as such, should not be used to exclude the disease in highly suspicious cases. Recently, a small case series described six dogs diagnosed with angiostrongylosis that had invalid *A. vasorum* lateral flow assays [132]. Dogs with a likely heavy burden of *A. vasorum* infection may have an invalid result due to control failure alongside a strongly positive test line. This was explained by the hook effect, also known as the postzone phenomenon, in which excess antigens and the formation of antibody–antigen immune complexes lead to a negative control result. In dogs with compatible clinical signs, repetition of the test with a diluted serum sample was suggested [132].

A further alternative for the diagnosis of canine angiostrongylosis is by means of molecular methods from fecal samples, blood, BAL fluid, and pharyngeal swabs [130]. A polymerase chain reaction (PCR) specifically amplifying DNA of *A. vasorum* was designed based on sequences of the internal transcribed spacer 2 (ITS‐2) regions of *A. vasorum*, *A. cantonensis*, *A. costaricensis*, and *Aelurostrongylus abstrusus* [26]. This was followed by the development of a quantitative polymerase chain reaction (qPCR) assay also targeting the ITS‐2 region and evaluated on both blood and fecal samples [27]. The clinical use of the qPCR test on BAL fluid has been described more recently in a small series of dogs diagnosed with angiostrongylosis [124]. In the study, all dogs had a positive qPCR on BAL, whereas only two of five dogs were positive when the Baermann technique was used, highlighting that qPCR on BAL fluid may represent a valuable diagnostic method, especially in acute infections. Larger prospective studies are needed to evaluate the sensitivity of qPCR on BAL.

Knowledge of the advantages and drawbacks of each technique, as well as the timing of detection of the infection, is important to choose the appropriate diagnostic method in each individual case. Table 2 provides a summary of the current diagnostic options for canine angiostrongylosis. Although the consequences of failing to diagnose *A. vasorum* infection can be worse than treating the infection empirically in an uninfected dog [4, 133], this practice should not encourage a decrease in investigations being performed. Rather, given the importance of detecting the disease from an individual and epidemiological point of view, all efforts should be made with the improved diagnostic capabilities to reach a definitive diagnosis.

**TABLE 2:** Summary of the current diagnostic options for canine angiostrongylosis.

Diagnostic test	Time of earliest detection	Sensitivity and specificity	Advantages	Disadvantages
Baermann technique	47–53 days pi [130]	Unknown	Morphologic differentiation from other lungworms	Collection and preservation of the sample Limited sensitivity due to intermittent shedding of L1 and false‐negative results during prepatency Length of time to obtain results Skilled staff required
Direct fecal smears	During patency	Sensitivity: 54%–61% [122]	In‐clinic and rapid diagnosis Inexpensive	Limited sensitivity Skilled staff required
Angio Detect (circulating antigen)	63–98 days pi [24]	Sensitivity: 84.6%–97.1% [24, 130, 131] Specificity: 98.9%–100% [24, 130, 131]	In‐clinic and rapid diagnosis Ease of obtaining the sample	Late detection of the infection Limited sensitivity (comparable to Baermann)
ELISA (circulating antigen)	35–77 days pi [24, 130]	Sensitivity: 95.7% [24] Specificity: 94% [24]	Quick diagnostic tool Possible detection of cases during prepatency Ease of obtaining a sample	Not readily available
ELISA (circulating antibodies)	13–42 days pi [25, 130]	Sensitivity: 85.7% [25] Specificity: 98.8% [25]	Quick diagnostic tool Detection of cases during prepatency Ease of obtaining the sample	False‐positive results in previously exposed dogs
BAL cytology	60 days pi [120]	Unknown	Possible detection before fecal larval shedding Morphologic differentiation from other lungworms Cytologic and microbiologic evaluation of the airways	Invasive technique Costly Not readily available
PCR in BAL	—	Unknown	Possible detection before fecal larval shedding Ability to detect low levels of pathogen DNA Specific species identification	Invasive technique Costly Not readily available
PCR in blood	From 13 to 70 days pi (inconsistent results) [130]	Unknown	Early detection of infection (during prepatency) Ease of obtaining the sample Ability to detect low levels of pathogen DNA Specific species identification	Low sensitivity High variation in results during patency
PCR in feces	55–77 days pi [130]	Unknown	Ability to detect low levels of pathogen DNA Specific species identification Suitable for preserved (frozen) samples	Collection of the sample Patent infections only High variation in results during patency

Abbreviations: BAL, bronchoalveolar lavage; L1, first‐stage larvae; PCR, polymerase chain reaction; pi, postinoculation.

### 4.4 Diagnostic Imaging

Thoracic radiography has historically been the mainstay of investigating thoracic disease. The intense immune response induced by *A. vasorum* eggs and migration of larvae throughout the pulmonary parenchyma causes variability in lung changes that can be appreciated radiographically [40, 57, 94, 134]. Severity of respiratory signs does not always correlate with imaging findings, as radiographic abnormalities have been documented even in patients without respiratory signs [57, 135]. Larval load and the timing of infection seem to be important factors related to the degree of pulmonary changes, with higher infectious loads causing earlier and more severe pulmonary changes [94, 136]. Although the radiographic findings associated with *A. vasorum* infection are not pathognomonic, the most characteristic radiologic change is a multifocal alveolar pattern, predominantly seen in the periphery, corresponding to pulmonary hemorrhage and nodules of inflammatory infiltrates as described histologically [136]. This distribution is not typical of other differentials for an alveolar pattern and, therefore, suspicion of canine angiostrongylosis should arise with this finding, especially in a young dog that lives in or has frequented an endemic area [4, 134]. The lung pattern varies over time, with an alveolar pattern and bronchial thickening appearing more commonly in early stages of the infection, while an interstitial pattern corresponding to areas of pulmonary fibrosis may persist after treatment and resolution of clinical signs [94, 136]. In experimentally infected dogs, the most pronounced radiographic changes were seen at the time of patency, and the severity of the radiographic changes progressed until 13 weeks after inoculation, with resolution of the alveolar pattern 9 weeks after treatment [94]. Vascular changes were observed in some cases, including enlarged pulmonary arteries and tortuous vessels suggestive of pulmonary hypertension due to thromboembolism and the potential presence of parasites. Pleural effusion or pleural fissures have also been described in naturally and experimentally infected dogs [40, 94].

Recently, advanced imaging techniques have come to the forefront in human and veterinary medicine, in many cases substituting thoracic radiography. Computed tomography (CT) provides higher‐resolution imaging compared with conventional radiography and is a sensitive method for detecting subtle pulmonary changes and producing a detailed assessment of the pulmonary vasculature, with potential detection of pulmonary thromboembolism. The CT findings of canine angiostrongylosis have been described in experimentally and naturally occurring infection [135, 136]. Diffuse‐to‐multicentric ground‐glass opacification, nodular patterns, increased lung attenuation affecting multiple lobes, and consolidating areas, especially in the ventral areas of the lung lobes, are documented CT features of infected dogs. Pleural fissures, suggestive of pleural effusion or pleuritis, were reported in experimentally infected dogs, but this was not identified in naturally infected dogs. Tracheobronchial lymphadenopathy was an inconsistent finding. Some of these CT findings are represented in Figure 3.

**FIGURE 3:**
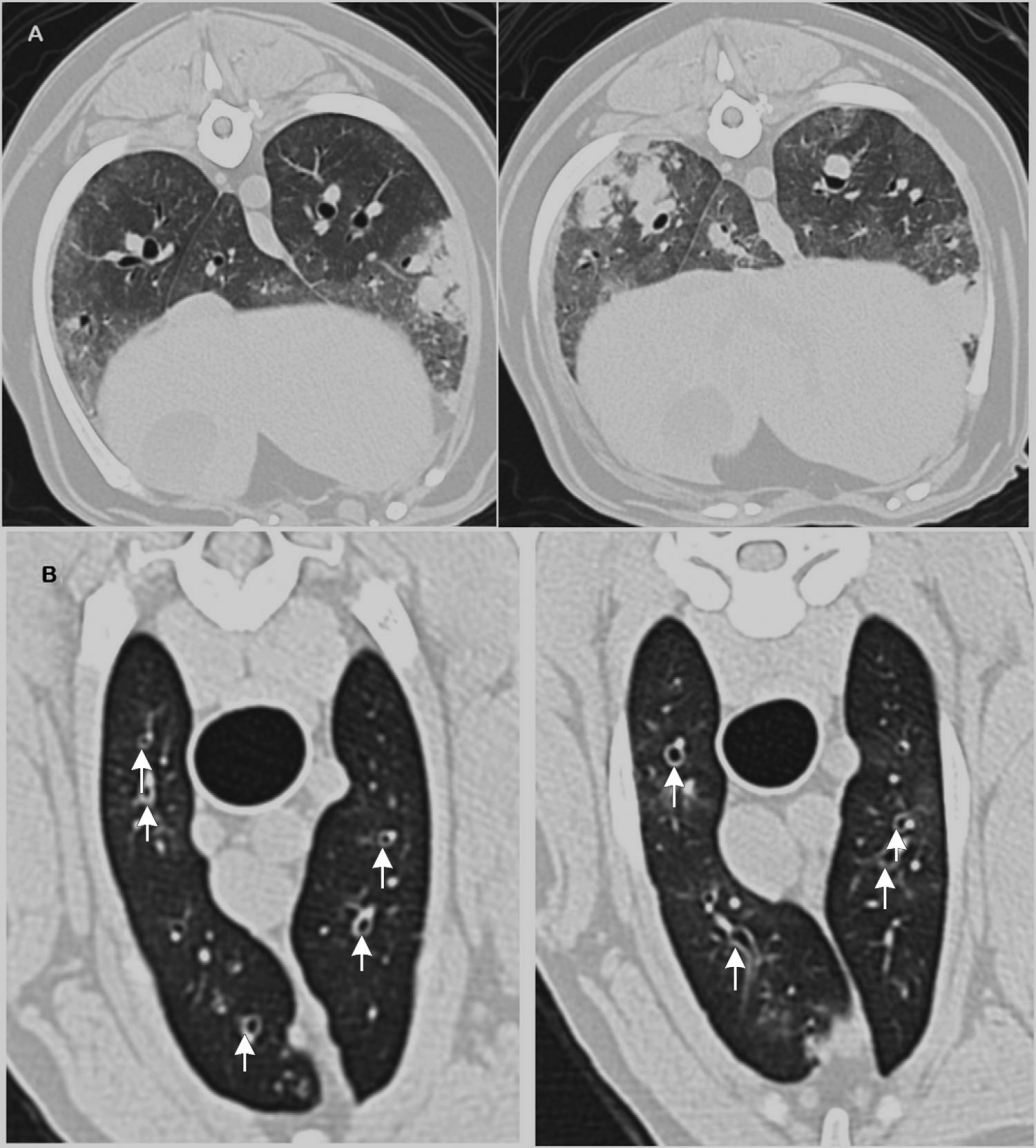
(A) CT scan image of the thorax of an 11‐year‐old neutered male Staffordshire Bull Terrier with multiple soft tissue attenuation masses with irregular margins and patchy areas of increased soft tissue attenuation in the pulmonary parenchyma. (B) CT scan image of the thorax of a 5‐year‐old neutered male Border Collie with generalized moderate bronchial pattern with bronchial wall thickening (white arrows). These CT features were present in two dogs diagnosed with *Angiostrongylus vasorum* infection. CT, computed tomography.

Magnetic resonance imaging (MRI) has become a valuable modality for assessing intracranial and spinal cord abnormalities. The MRI findings of dogs with neurologic signs associated with *A. vasorum* infection are mostly consistent with cerebral or spinal hemorrhage, characterized by isointense signal in T1‐weighted images, hyperintensity in T2‐weighted images, and hypointensity in gradient echo images [98, 101, 102] Although a vascular event is usually the presumed cause for the neurologic signs in these patients, inflammatory meningeal disease has also been observed in the MRI findings of a dog in which *A. vasorum* larvae were identified in CSF cytology [105]. CSF analysis has been rarely reported in the literature, partly because the increased risks of bleeding in these patients preclude a CSF sample from being obtained; therefore, CSF findings are not well documented. Typically, CSF analysis is compatible with a hemorrhagic CNS event [98, 102]; however, a recent case report documented eosinophilic pleocytosis associated with *A. vasorum* infection in a dog [101].

Echocardiography is a noninvasive method for the diagnosis of pulmonary hypertension and the best method to assess cardiac function. Echocardiographic findings described in canine angiostrongylosis include right atrial and ventricular dilation, systolic septal flattening, reduced left ventricular size, dilated pulmonary trunk, changes in pulmonary flow profiles, and tricuspid and pulmonary insufficiency [52]. In a recent retrospective study, the prevalence of moderate‐to‐severe pulmonary hypertension was identified in up to 35% of cases with an overall prevalence of 58.8% [57], contrasting with previous studies that found a lower presence of pulmonary hypertension [51, 55, 94]. The authors of this recent work chose to evaluate other echocardiographic parameters (e.g., acceleration time/ejection time ratio, main pulmonary artery/aorta ratio, pulmonary flow pattern) in the absence of tricuspid regurgitation to identify pulmonary hypertension, suggesting that this condition may have been underestimated in *A. vasorum* infection. Considering this, investigations focused on determining the presence of pulmonary hypertension are indicated to optimize the therapeutic management of these patients.

In recent years, point‐of‐care ultrasound (POCUS) has become a readily available imaging modality and has been increasingly incorporated as part of the initial assessment of critical patients presenting to the emergency room. Thoracic POCUS is a noninvasive procedure that can be used rapidly and reliably to identify and diagnose pulmonary disease [137]. In an emergency setting, thoracic POCUS is of great value in assessing dyspneic patients because it requires minimal handling and may allow differentiation of cardiac versus pulmonary disease; additionally, it can be used to assess for pleural disease. Small (2–25 mm), solid, subpleural pulmonary nodules have been described using ultrasonography in canine angiostrongylosis [138, 139]. The use of thoracic ultrasonography has been recently evaluated in 26 privately owned dogs infected with *A. vasorum* presenting with respiratory distress [139]. The study showed how thoracic POCUS can be a useful tool for the diagnosis of canine angiostrongylosis with a high sensitivity and specificity. However, the decreased use of prophylactic drugs against *A. vasorum* infection in the selected population may have added to the relatively high disease prevalence, which likely enhanced the positive predictive value for this particular study population. Furthermore, the study only included young patients (<2 years of age), reducing the possibility of finding a neoplastic process as the cause for a nodular lung pattern. Larger prospective clinical studies are needed to further assess the accuracy of thoracic POCUS for the diagnosis of canine angiostrongylosis.

## 5 Anthelmintic and Supportive Therapy

Dogs infected with *A. vasorum* can suffer from several potential complications if left untreated, stressing the importance of a rapid diagnosis and the early use of directed anthelmintic drugs. Management of canine angiostrongylosis is based on the use of either a macrocyclic lactone or a benzimidazole anthelmintic agent alongside supportive care (European Scientific Counsel Companion Animal Parasites guidelines). Treatment of underlying complications, such as coagulopathies and respiratory distress, is essential for more severe cases.

Several anthelmintic protocols have been reported to be effective against *A. vasorum* infection (Table 3). Traditionally, levamisole, ivermectin, and fenbendazole have been used to treat canine angiostrongylosis with good, reported efficacy [109, 140, 141]. However, the use of these drugs has diminished, given the concerns derived from levamisole use [141] and the documented toxicity of ivermectin in susceptible Collie breeds with the ABCB1 gene (MDR1) mutation, and safer drugs have replaced their use. Fenbendazole, a broad‐spectrum benzimidazole drug, used at various dosages (20–50 mg/kg for 5–21 days), has been a popular option. Although the optimal dose and duration of therapy have not been determined, a recent survey showed that fenbendazole remains one of the most common treatments used by veterinary practitioners in the United Kingdom, despite not being licensed for canine angiostrongylosis in the country [142]. Given the lack of an established protocol, dogs managed with fenbendazole may potentially receive a suboptimal treatment [142]. The reason for this preference for drug choice among clinicians is unclear, especially when newer drugs from the group of macrocyclic lactones, such as milbemycin oxime and moxidectin, have been developed and licensed for their use in canine angiostrongylosis. Several studies have demonstrated these drugs’ efficacy against *A. vasorum* in experimentally and naturally infected dogs [143, 144]. Willesen et al. conducted a randomized, blinded, controlled multicenter field study to evaluate the efficacy of a single spot‐on application of combined moxidectin (2.5–6.25 mg/kg) and imidacloprid (10–25 mg/kg) and compared it with daily fenbendazole (25 mg/kg for 3 weeks) in dogs naturally infected with *A. vasorum* [143]. The spot‐on formulation was found to be safe and effective against both immature and mature stages of *A. vasorum*. The study showed high therapeutic efficacy with no significant differences between the two treatment protocols (85.2% and 91.3%, respectively). Similarly, a previous study using milbemycin oxime administered orally at 0.5 mg/kg on a weekly basis for four treatments reported an efficacy of 84.8% in dogs experimentally infected with *A. vasorum* [144]. The primary outcome measurements evaluated in these studies were different, and therefore, a direct comparison between the efficacy of imidacloprid/moxidectin and milbemycin oxime cannot be made.

**TABLE 3:** Anthelmintic protocols reported to be effective against *Angiostrongylus vasorum* infection in dogs.

Drug	Dose	Route and frequency of administration
Imidacloprid/moxidectin [143]	2.5–6.25 mg/kg moxidectin 10–25 mg/kg imidacloprid	Single spot‐on dose (repeated after 4 weeks if required)
Moxidectin/sarolaner/pyrantel^a^ [118]	0.024–0.048 mg/kg moxidectin 1.2–2.4 mg/kg sarolaner 5–10 mg/kg pyrantel	Single oral dose (repeated after 4 weeks if required)
Milbemycin‐oxime [144]	0.5 mg/kg	Orally once weekly for 4 weeks
Fenbendazole [39, 143]	20–50 mg/kg	Orally once daily for 5–21 days
Ivermectin [140, 147]	200–400 µg/kg	Subcutaneously every 3 weeks for two to four treatments

^a^ Licensed for treatment in the European Union.

In coagulopathic patients, specific treatment to target the altered hemostasis is indicated. Abnormalities in one or more aspects of the coagulation pathways may be involved, including factor deficiencies, hyperfibrinolysis, thrombocytopenia, and other previously discussed mechanisms. Choice of treatment can be guided by viscoelastic methods when available. Fresh frozen plasma (FFP) (10–20 mL/kg) is used in hypocoagulable patients with coagulation factor deficiencies, whereas cryoprecipitate, a source of concentrated fibrinogen, may be preferable instead of high doses of FFP in patients with hypofibrinogenemia [95]. Whole blood or packed red blood cell transfusion may be required if symptomatic anemia is present while the infection is treated with an appropriate anthelmintic.

The use of antifibrinolytic drugs should be considered in patients with documented primary hyperfibrinolysis [41, 44, 61]. Tranexamic acid (TXA) and epsilon‐aminocaproic acid are the most common antifibrinolytic agents used in veterinary medicine; however, there are no reports describing the use of epsilon‐aminocaproic acid in canine angiostrongylosis; as such, TXA is currently the antifibrinolytic drug of choice. Plasmin generation is inhibited by these drugs due to their competitive binding to the C‐terminal lysine site on plasminogen, and as a result, plasminogen is prevented from binding fibrin, thereby reducing fibrinolysis [61]. A retrospective study showed that dogs with *A. vasorum* infection and evidence of hyperfibrinolysis on ROTEM responded successfully to TXA with a documented decrease in maximum clot lysis [44]. Although a consensus for the treatment of hyperfibrinolysis in dogs is yet to be established, the current recommended dose of TXA in dogs with active bleeding is 15 mg/kg administered as a slow infusion (over 15–20 min) every 8 h until hemorrhage resolves [145]. TXA dosages have been extrapolated from human medicine and pharmacokinetic studies conducted in healthy dogs [145]. However, there is some evidence that dogs are relatively hyperfibrinolytic compared with people [80], suggesting the need for higher doses of TXA in dogs with hyperfibrinolysis [84, 87]. In particular, some dogs with *A. vasorum*‐induced hyperfibrinolysis required doses >20 mg/kg to normalize the fibrinolytic activity [44, 45].

Theoretically, antifibrinolytic agents increase the risk of thromboembolic complications; as such, antifibrinolytic therapy is generally avoided in the presence of secondary hyperfibrinolysis, in which a prothrombotic state is responsible for the upregulation of the normal fibrinolytic system [64]. Dogs infected with *A. vasorum* may be hypercoagulable [42]; therefore, empirical use of these drugs should be carefully considered, balancing the risks of bleeding and thrombosis in each individual case. Viscoelastic testing serves as a valuable tool in determining the correct use of antifibrinolytic drugs and in guiding the optimal duration of treatment. Anecdotally, some authors advise continuing with TXA for 72 h after resolution of hemorrhage [95], although in most cases bleeding usually resolves after appropriate anthelmintic therapy. Oral administration of TXA has a delayed onset of action compared with intravenous administration, but it provides a prolonged antifibrinolytic effect, and therefore an intravenous loading dose followed by 15–20 mg/kg orally every 8 h has been proposed [145]. Vomiting and nausea are known adverse effects of TXA, especially when high doses (20 mg/kg) are used or when the drug is administered too quickly intravenously. Maropitant, a neurokinin 1 receptor antagonist used as pretreatment before TXA infusion, has been shown to be effective in preventing these adverse effects [146].

Thrombocytopenia and thrombocytopathia are rarely the main cause of bleeding in dogs infected with *A. vasorum*. Although thrombocytopenia is a frequent finding in these patients, it is usually only mild to moderate, and correction of the concurrent hemostatic disorder causing the consumptive thrombocytopenia, as well as administration of an appropriate anthelmintic treatment, will likely normalize platelet count. In patients that develop acquired von Willebrand syndrome secondary to angiostrongylosis, therapy with FFP, cryoprecipitate transfusion, or desmopressin may be of benefit, although, as with thrombocytopenia, specific treatment is rarely required in these cases. If there is evidence that the cause of hemorrhage is due to immune‐mediated thrombocytopenia, as reported in a minority of cases, and no other hemostatic alterations are identified, immunosuppressive doses of corticosteroids may be considered [69, 73].

Occasionally, worsening of the respiratory signs after administration of anthelmintic treatment may occur due to the presence of dead worms in the lower airways and pulmonary parenchyma and the induced inflammatory response. Pretreatment with corticosteroids has been used to reduce the risk of anaphylaxis and minimize the immune response induced by the death of mature and immature stages of *A. vasorum* after anthelmintic therapy has been initiated [26, 71, 124]. This approach is likely carried over from the recommendations established for the treatment of *D. immitis* or other pulmonary parasites of veterinary species, such as *Dictyocaulus viviparus*, in which anti‐inflammatory doses of glucocorticoids are used to mitigate the treatment‐associated complications. However, there is currently a lack of evidence for this use in canine angiostrongylosis. Corticosteroid use in these cases seems to be heavily related to individual clinician preference and experience.

Congestive heart failure and pulmonary hypertension are other complications that may occur because of *A. vasorum* infection [51, 52]. Appropriate therapy for congestive heart failure, such as diuretics, positive inotropic drugs (e.g., pimobendan), and angiotensin‐converting enzyme inhibitors, is indicated to minimize the effects of cardiac dysfunction. Sildenafil is used for moderate‐to‐severe pulmonary hypertension. In a small retrospective study, the use of sildenafil was not significantly associated with improved survival times compared with control dogs with pulmonary hypertension secondary to *A. vasorum* infection [51]. However, the lack of a standardized approach and the small sample size in the study prevent clear conclusions from being drawn, and further evaluation of the use of sildenafil in this population is warranted. Further supportive treatment should be provided according to the needs of each individual case. Oxygen therapy for the respiratory‐compromised patient, intravenous fluid therapy and supplementary feeding to restore fluid deficits, and appropriate nutrition are essential. Parasitic and bacterial coinfections have been reported in the literature [16, 124, 147, 148]. Antibiosis should be implemented using contemporaneous clinical guidelines if a clinically relevant concurrent bacterial pneumonia is identified on BAL fluid evaluation [149]. The prevalence of concurrent bacterial infections in canine angiostrongylosis is unknown, as microbiological assessment of BAL fluid may not be deemed necessary in cases where the diagnosis is reached by noninvasive tests. Without a clinical supportive indication, empirical antimicrobial therapy should be avoided.

Therapeutic effectiveness of anthelmintic treatment is usually apparent with the resolution of clinical signs. Confirmation of negative serologic or fecal tests after therapy is rarely performed in the clinical setting and is probably not necessary if the clinical signs resolve. Successful clearance of infection after treatment has been evaluated experimentally [130]. Fecal tests were the first to detect negative results after treatment: 1 week for the modified Baermann technique and 2 weeks for PCR. These tests, however, have a high variability in results during infection due to the intermittent shedding of L1 and may therefore not be the most reliable for confirmation of parasite clearance. Circulating antigen was no longer detected 5 weeks after treatment, and antibodies were undetectable within 3–9 weeks after treatment. Serologic methods may therefore provide more certainty of successful parasite clearance [130]. These results, however, were obtained from experimental infection of a homogenous cohort of dogs, and outcomes in naturally infected populations might differ.

## 6 Prognosis

The prognosis of canine angiostrongylosis is variable and highly dependent upon the clinical presentation. Multiple cases with fatal outcomes have been reported. However, publication bias could also explain this via increased reporting of more severe cases, especially in areas previously unaffected by *A. vasorum* [3, 18, 19]. Although the outcomes of less severe clinical cases may be favorable, those presenting with respiratory compromise or with coagulopathies do likely carry poorer prognoses [41, 42, 43, 51]. In a large retrospective study, 50 of 65 dogs (76.9%) with evidence of bleeding survived to hospital discharge, contrasting with 109 of 115 dogs (94.8%) without bleeding diathesis [42]. Conversely, in another retrospective study with a much smaller group of dogs, 14 of 21 dogs (67%) survived to discharge, both those with clinical signs of bleeding and without [44]. A favorable outcome was reported in an older study that included 53 dogs with mild‐to‐moderate clinical signs, all of which survived [143]. Yet the same group reported that 3 of 24 infected dogs (12.5%) at their referral hospital died [4]. This is likened to data from a study by Chapman et al. in which 3 of 23 infected dogs (13%) died [39]. Early recognition of coagulopathic dogs and prompt administration of targeted therapy addressing the hemostatic dysfunction seem to be essential. Of note, some dogs with bleeding tendencies may only present with neurologic signs, also often carrying a poor prognosis [42].

## 7 Future Aspects in Research

As discussed in this review, many aspects of canine angiostrongylosis are currently being investigated. Of relevance, the genomic sequence of *A. vasorum* has been recently determined [75]. A better understanding of the parasite genome may lay the foundations for the development of alternative prophylactic measures for the disease (e.g., vaccination). Prophylactic therapies have been developed in recent years [114, 115, 116, 117, 118], and these drugs have been proven to be highly effective when administered on a monthly basis, preventing migration of L1 through the pulmonary parenchyma and its consequent lung inflammation and damage. However, the regular administration required to maintain protection has raised concerns for possible anthelmintic resistance [150] and the potential environmental impact of drug residues.

Additionally, dogs belonging to noncompliant owners will remain at risk of infection. Development of a vaccine against *A. vasorum* is of great interest as this would overcome the limitations of the current preventive strategies and provide a possible control measure for infection in wildlife [75]. Moreover, the currently available genomic data are valuable for the development of new genomic markers for *A. vasorum*. The widely used marker ITS‐2 has often produced sequencing errors [151]; therefore, the new microsatellite regions identified in this recent work should be further explored [75].

Because of its fatal consequences, understanding the exact underlying causes of bleeding in canine angiostrongylosis is one of the most important aspects being investigated. Recent experimental studies have shed light on possible factors involved in the disruption of the host coagulation [46, 47, 48, 75]; however, while significant advancements have been made, the precise interactions between the parasite and the hemostatic system require further evaluation. This experimental work will eventually lead to new areas of research and be used to identify potential drug targets and vaccine candidates. The genomic data have allowed investigators to evaluate genetic variations within *A. vasorum* populations. It is possible that the reported genetic diversity of *A. vasorum* may play a role in the pathogenicity of the parasite [152], which could be one of the reasons why some dogs develop more severe disease than others. From a clinical point of view, with new insights regarding the pathologic mechanisms leading to hemostatic dysfunction, larger studies that evaluate the coagulation status of dogs naturally infected with *A. vasorum* and the frequency of these abnormalities are needed to improve and provide tailored therapy for the individual patient.

*Angiostrongylus vasorum* is a highly pathogenic parasitic disease that continues to draw the attention of the veterinary community due to its geographic expansion. Perceived underestimation of the disease and difficulties reaching a final diagnosis have led to the development of improved diagnostic tests. Understanding the pathophysiology of coagulopathies associated with *A. vasorum* infection continues to improve, but some aspects remain undiscovered. Recent insights into the pathogenesis of *A. vasorum* and genetic studies have provided new areas of focus, which will hopefully allow for an increasingly targeted approach to management.

## Author Contributions

**Iris Elgueta**: conceptualization, investigation, visualization, writing – original draft, writing – review and editing. **Daniel Lewis**: conceptualization, investigation, visualization, writing – original draft, writing – review and editing. **Eric R. Morgan**: conceptualization, investigation, visualization, writing – original draft, writing – review and editing. **Jenny Helm**: conceptualization, investigation, visualization, writing – original draft, writing – review and editing.

## Ethics Statement

No ethical approval was required as this is a review article with no original research data.

## Conflicts of Interest

The authors declare no conflicts of interest.

## Endnote

1 Angio Detect test, IDEXX Laboratories, Westbrook, ME.
